# Dissolution behaviour of radiocaesium-bearing microparticles released from the Fukushima nuclear plant

**DOI:** 10.1038/s41598-019-40423-x

**Published:** 2019-03-05

**Authors:** Taiga Okumura, Noriko Yamaguchi, Terumi Dohi, Kazuki Iijima, Toshihiro Kogure

**Affiliations:** 1 Department of Earth and Planetary Science, Graduate School of Science, The University of Tokyo, 7-3-1 Hongo, Bunkyo-ku, Tokyo 113-0033 Japan; 2 Institute for Agro-Environmental Sciences, NARO, 3-1-3 Kannondai, Tsukuba, Ibaraki 305-0864 Japan; 3 Fukushima Environmental Safety Center, Sector of Fukushima Research and Development, Japan Atomic Energy Agency, 10-2 Fukasaku, Miharu-machi, Tamura-gun, Fukushima 963-7700 Japan

## Abstract

Radiocaesium-bearing microparticles (CsMPs) composed of silicate glass were released by the Fukushima Daiichi Nuclear Power Plant accident in March 2011. Since CsMPs contain a high concentration of radiocaesium, their dynamics and fate in the environment are urgent issues to be investigated. Here, we show that CsMPs are dissolved by weathering in the environment and that their radioactivity is more rapidly decreased by dissolution than the physical decay of radiocaesium. We conducted dissolution experiments with CsMPs in pure water that absorbed CO_2_ from the atmosphere and in artificial seawater at several temperatures. The dissolution progress was monitored by the decrease in the ^137^Cs radioactivity in CsMPs, and the dissolution rate was estimated. The activation energy for the dissolution of CsMPs was estimated to be 65 and 88 kJ/mol and the dissolution rate at 13 °C (approximate annual mean temperature in Fukushima City) was 0.014 and 0.140 μm/y for pure water and seawater, respectively, assuming that radiocaesium is uniformly distributed in spherical CsMPs. The shapes of the CsMPs dissolved in pure water were considerably altered, suggesting that the dissolution proceeded without maintaining the geometry. Tin oxide and iron oxide nanoparticulates formed on the surfaces. Such features were similar to those observed in CsMPs recently collected in Fukushima Prefecture, indicating that weathering dissolution of CsMPs is also occurring in the environment. For the CsMPs dissolved in seawater, a crust of secondary Mg- and Fe-rich minerals was formed, and the glass matrix inside the crust decreased, creating space between the crust and the glass matrix.

## Introduction

A significant amount of radiocaesium (^134^Cs and ^137^Cs) was released by the Fukushima Daiichi Nuclear Power Plant (FDNPP) accident in March 2011, resulting in radiation contamination around the FDNPP. Two forms of radiocaesium were released from the FDNPP. The gaseous form, which is considered to have occupied most of the released radiocaesium, was transported with sulfate aerosols in the atmosphere and deposited on the ground by rain^1–3^, and this form of radiocaesium is soluble in water and strongly fixed by specific minerals in soil, such as weathered biotite^4,5^. The other form is an insoluble particulate with a high concentration of radiocaesium, and this form was directly emitted by the damaged reactors. Adachi *et al*.^6^ discovered spherical radioactive microparticles with diameters of 2.0–2.6 µm and ^137^Cs of 0.7–3 Bq in a single particle in aerosol filters collected at Tsukuba, Japan, 170 km southwest from the FDNPP. These radiocaesium-bearing microparticles are termed “CsMPs.” Abe *et al*.^7^ reported that these CsMPs contain several ^235^U fission products, confirming that the CsMPs were formed in the reactors. Yamaguchi *et al*.^8^ found similar CsMPs on ground-cover material for agriculture and leaves in Fukushima Prefecture. They used transmission electron microscopy (TEM) to determine that the main component in the CsMPs is silicate glass in which Cl, K, Fe, Zn, Rb, Sn, and Cs are the major dissolved constituent elements. Kogure *et al*.^9^ characterized the inner structure of the CsMPs using scanning transmission electron microscopy (STEM) equipped with energy-dispersive X-ray spectroscopy (EDS) with ultra-high detection efficiency. They determined the quantitative chemical compositions and elemental distributions in CsMPs and found caesium enrichment near the surface. Yamaguchi *et al*.^10^ discovered non-spherical CsMPs with constituent elements and inner structures that are different from those reported in previous works^9,11,12^. In addition, Satou *et al*.^11^ reported a different type of radiocaesium-bearing particles that are larger than CsMPs. The property and fate of CsMPs have been recently investigated in relation to incineration of radiation-contaminated waste^13^. The radioactivity of ^137^Cs in CsMPs, which is a few Bq in a single particle, is far higher than that of radiocaesium-sorbing clay minerals, which contain only 10^−3^ to 10^−2^ Bq of ^137^Cs in a 50 µm particle^14^. Therefore, internal exposure caused by inhalation of CsMPs is a concern. The impact of CsMPs on the human respiratory system is also a concern because of their size, i.e., a few microns, which is similar to that of PM_2.5_. Hence, the dynamics and fate of CsMPs in the environment urgently need to be investigated. However, such properties of CsMPs have not been reported because collecting and separating CsMPs from other particulates are very laborious processes^8,15,16^.

When Adachi *et al*.^6^ first reported CsMPs, they demonstrated that CsMPs do not dissolve in water like soluble aerosol particles, such as halides and sulfates. Accordingly, CsMPs have been frequently considered insoluble particles. However, CsMPs are mainly composed of silicate glass, which can be slowly dissolved in aqueous solutions. Hence, CsMPs are not truly insoluble, and their solubility should be considered in their long-term behaviour. The purpose of this study is to estimate the residence time of CsMPs in water through dissolution experiments. Although this estimation would be more accurate if synthetic bulk glass with similar chemical compositions to those of CsMPs was used for the dissolution experiments, such glass has not been synthesized to date^9^. In the present study, we collected CsMPs from non-woven fabric cloth (NWC) laid on a vegetable field in Fukushima Prefecture using a recently reported method^15^. Moreover, we investigated the dissolution process of CsMPs by analysing the CsMPs before and after dissolution experiments using electron microscope techniques.

## Results

### Dissolution rate of CsMPs

We estimated the dissolution rate of CsMPs in pure water and artificial seawater using two different experiments, namely, the single CsMP experiment and multiple CsMP experiment. In the single CsMP experiment, individual CsMPs were isolated from the NWC, identified using scanning electron microscopy (SEM), and then immersed in pure water or seawater under several temperature conditions. In the multiple CsMP experiment, fragments of the NWC with several CsMPs identified by imaging plate (IP) autoradiography were immersed in pure water or seawater. The pH of the pure water and seawater during the dissolution experiments was always approximately 5.2 and 8.3, respectively. The pure water was slightly acidic because it absorbed CO_2_ from the atmosphere before the experiments. The change in the radioactivity of ^137^Cs in the CsMPs was monitored before, during, and after immersion, and the results are summarized in Tables S1–S4. The radioactivity of ^137^Cs in the CsMPs decreased with increasing immersion time and temperature, which indicated that the CsMPs slowly and gradually dissolve in aqueous solutions in the environment. Furthermore, the dissolution rates in seawater were much faster than those in pure water at the same temperature.

Next, we converted the decrease in radioactivity into the CsMP dissolution rate or decrease in CsMP radii using the following method. Assuming that the CsMPs are spherical in shape and that radiocaesium is uniformly distributed within them, the CsMP radii (*r*) used in the dissolution experiments was calculated by the following equation:

1 $$r=3\sqrt{\frac{3R}{4\pi \rho N}}$$

where *R* (Bq) is the radioactivity of the specimen, *ρ* (Bq/m^3^) is the ^137^Cs radioactivity per unit volume of CsMPs, and *N* is the number of CsMPs in each experiment. In the case of the single CsMP experiment, the original radius, *r*_0_, could be measured by SEM observations. Since *N* = 1 and the initial radioactivity, *R*_0_, was also known, *ρ* could be obtained from eq. (1). Thus, the radii of the CsMPs (*r*) after incubation could be obtained from the radioactivity *R* at that time. Note that the CsMPs designated PS120-3, PS90-1, and PS90-2 for the experiments with pure water were not examined by SEM; their radii, *r*, were calculated by assigning the average *ρ* value of 0.327 Bq/μm^3^ from the other five CsMPs (PS120-1, PS120-2, SS90-1, SS90-2, and SS90-3) as the *ρ* value for these three CsMP samples. In the case of the multiple CsMP experiments, *r*_0_, *ρ*, and *N* were unknown. Thus, *ρN* was obtained from the original radioactivity *R*_0_ by using the average *r*_0_ values of 1.16 μm from the five CsMPs for the single CsMP experiment. By using the obtained *ρN* values, the radii, *r*, after incubation could be obtained from the radioactivity *R*. The radii of the CsMPs, which were determined based on the decrease in radioactivity using eq. (1), are shown in Tables S1–S4 as *r*_h_. Note that Satou *et al*.^11^ reported that the relationship between ^137^Cs radioactivity *R* (Bq) and volume *V* (cm^3^) is *R* = 2 × 10^16^ × *V*^1.43^, but we did not use this equation because their data widely varied within the radioactivity range found in this study (0.86–3.93 Bq for the CsMPs used in the single CsMP experiment).

Next, the change in the volume of the CsMPs per unit time (d*V*/d*t*), which was assumed to be proportional to the surface area, was expressed by the following equation:

2 $$\frac{dV}{dt}=-k\cdot 4\pi {r}^{2}$$

where *k* (m/s) is the rate of the decrease in the radius of the CsMPs. Equation (2) can be transformed as follows by using *dV* = 4*πr*^2^·*dr*:

3 $${r}_{0}-r=kt$$

**Figure 1:**
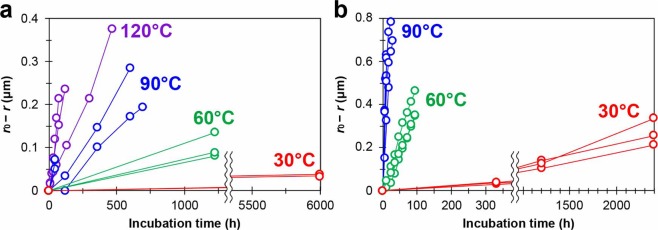
Decrease in the CsMP radius (*r*_0_ − *r*) versus incubation time, assuming homogeneous distribution of Cs in the CsMPs. (**a**) Pure water. (**b**) Seawater.

Using eq. (3) and the *r*_h_ values shown in Tables S1–S4, the decreased CsMP radii (*r*_0_−*r*) are shown as a function of incubation time (*t*) in Fig. 1. The decreased CsMP radii were approximately proportional to the incubation time, and the rates of decrease were much faster in seawater than pure water. The CsMP dissolution rates could be obtained as the rate of decrease in the radius (*k*) from the slopes in the figure.

**Figure 2:**
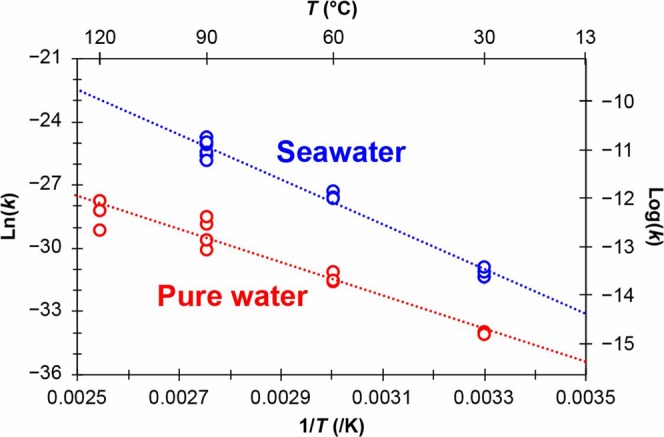
Arrhenius plot of the logarithm of *k* (rate of decrease in the radius of CsMPs; m/s) versus the reciprocal temperature 1/*T*, assuming homogeneous distribution of Cs in the CsMPs.

Figure 2 shows the Arrhenius plot (logarithm of *k* versus reciprocal temperature, 1/*T*) constructed from the *k* values obtained from the slopes in Fig. 1. The linearity of the Arrhenius plot (R^2^ = 0.93 and 0.98 for pure water and seawater, respectively) suggests that the assumptions mentioned above are valid. From the slope of the Arrhenius plot, the activation energy was calculated to be 65 and 88 kJ/mol for pure water and seawater, respectively. Additionally, the estimated *k* values were plotted against temperature, *T*, in Fig. S1. According to this figure, the *k* values at 13 °C (approximate annual mean temperature in Fukushima City^17^) were 0.014 and 0.140 μm/y for pure water and seawater, respectively. This result indicates that the ^137^Cs radioactivity of a CsMP with a radius of 1.16 µm (average radius in this study) would decrease by half after approximately 17.1 and 1.7 years in pure water and seawater, respectively, without considering the physical decay of radiocaesium.

These dissolution rates derived from the experiments inevitably contain several sources of error. For instance, the dissolution at a high temperature may possibly proceed differently from that at a low temperature. Since we are interested in the dissolution at a temperature in the environment, it may be more reasonable to estimate the dissolution rates at temperatures below 30 °C only from the experimental results at 30 and 60 °C. In this case, the activation energy was estimated 73 and 101 kJ/mol, and *k* values at 13 °C were 0.009 and 0.088 µm/y for pure water and seawater, respectively. We should keep in mind that the estimated dissolution rate of CsMPs has such uncertainty.

Although the above estimation was based on the assumption of a homogeneous Cs distribution in CsMPs, some CsMPs were reported to have a radial distribution of Cs with higher concentrations near the surface^9,18^. If the CsMPs used in this study also had such radial distribution of Cs, the dissolution rate calculated above might be overestimated. Therefore, the dissolution rate was recalculated assuming a radial distribution of Cs, such as that in the CsMP designated P6-6 in the previous research^9^. (See the supplementary information for the detailed calculation.) The activation energy was 65 and 92 kJ/mol for pure water and seawater, respectively, and the rate of decrease in the radius of the CsMPs was estimated to be 0.007 and 0.058 μm/y for pure water and seawater, respectively, at 13 °C. Accordingly, the dissolution rate of the CsMPs was approximately half the value calculated based on a homogeneous Cs distribution. To our knowledge, the CsMP designated P6-6 has the strongest tendency of radial distribution of Cs among the CsMPs analysed thus far. Therefore, the dissolution rates of CsMPs can be predicted to be between the values calculated assuming homogeneous and inhomogeneous distribution of Cs.

### Structural characterization of CsMPs in the dissolution experiment

**Figure 3:**
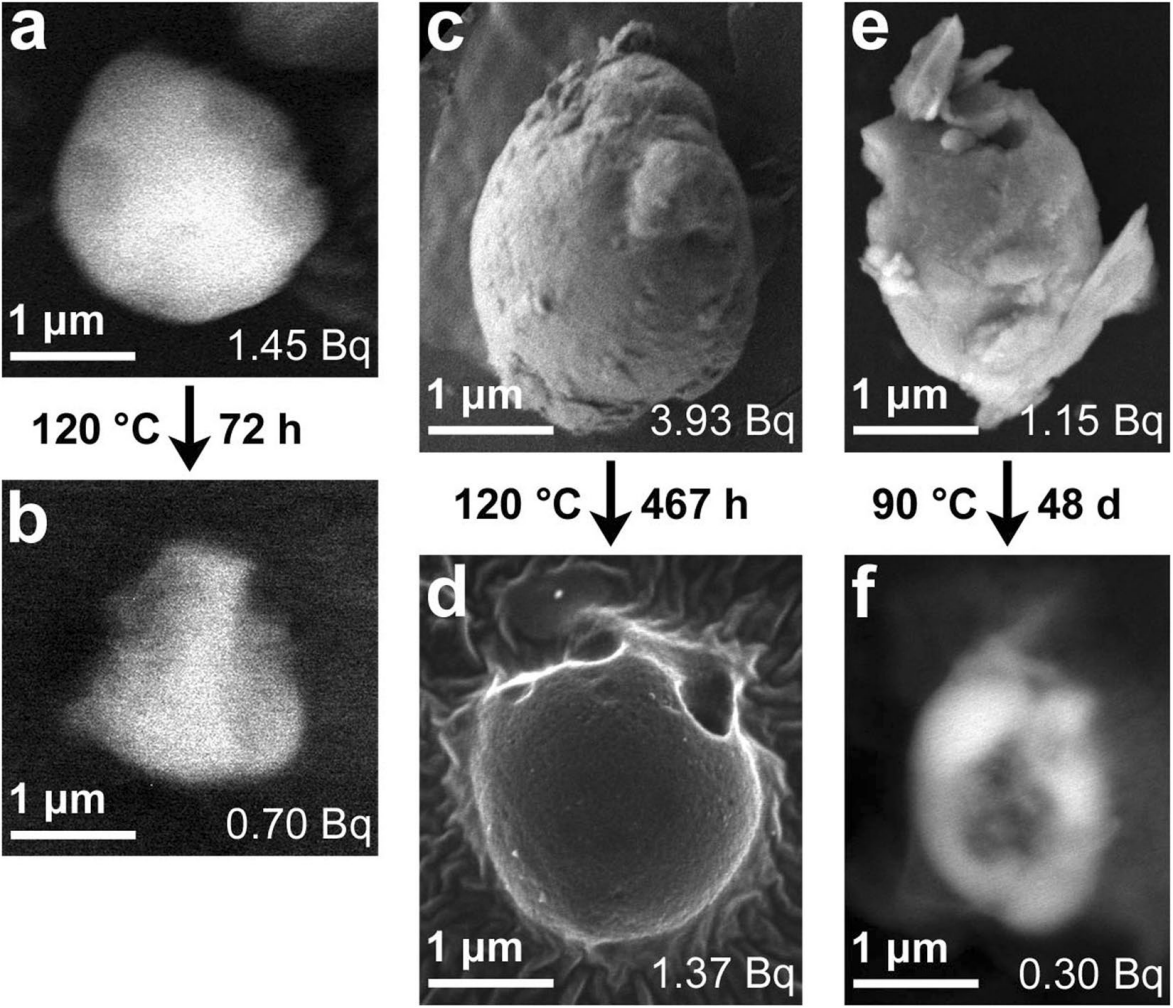
SEM images of PS120-1 (**a**,**b**), PS120-2 (**c**,**d**), and PS90-3 (**e**,**f**) before (**a**,**c**,**e**) and after (**b**,**d**,**f**) the dissolution experiment with pure water. The ^137^Cs radioactivity of the particles is also shown in the bottom-right of the images. The images in (**a**,**b**,**f**) were taken using back-scattered electrons at an accelerating voltage of 15 kV because the particles were buried in organic material, while the other images were taken using secondary electrons at an accelerating voltage of 2 or 5 kV.

The CsMPs before and after the single CsMP experiment were characterized using SEM to examine their structural changes during dissolution. We succeeded in collecting three and two CsMPs from solution after the experiments with pure water and seawater, respectively. The other CsMPs possibly split into several fragments during dissolution and could not be collected from the solutions. In the case of pure water, the SEM micrographs clearly showed that the CsMPs decreased in size after the dissolution experiment, indicating that the decrease in the radioactivity of ^137^Cs from the CsMPs was owing to the dissolution of the glass matrix and not to selective elution of caesium from the glass (Fig. 3). However, the shapes of the CsMPs were considerably altered, suggesting that the dissolution proceeded without maintaining the geometric shape. The SEM image taken with back-scattered electrons (BSE) of PS90-3 after dissolution showed a large hole in the centre of the particle (Fig. 3f).

**Figure 4:**
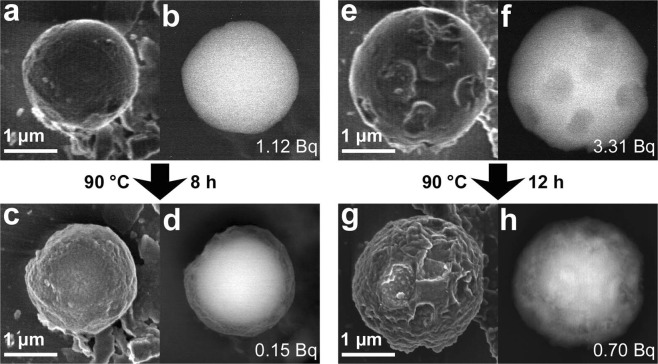
SEM images of SS90-1 (**a**–**d**) and SS90-2 (**e**–**h**) before (**a**,**b**,**e**,**f**) and after (**c**,**d**,**g**,**h**) the dissolution experiment with seawater. The images in (**a**,**c**,**e**,**g**) were taken using secondary electrons at an accelerating voltage of 2 or 5 kV, while the other images were taken using back-scattered electrons at an accelerating voltage of 15 or 20 kV. The ^137^Cs radioactivity of the particles is also shown in the bottom-right of the back-scattered electron images.

Meanwhile, the size of the CsMPs dissolved in seawater did not change despite the decrease in the ^137^Cs radioactivity (Fig. 4). However, the surface became rough after dissolution, especially that of SS90-2 (Fig. 4c,g). Furthermore, the surface became darker in the BSE image (Fig. 4d,h).

After dissolution, these CsMPs were processed into electron-transparent thin films by a focused ion beam (FIB) and subjected to STEM-EDS analysis. EDS spectra were acquired from the centre of the CsMPs (Fig. S5), and quantification was performed using these spectra (Table S5). The results confirmed that the CsMPs used in this study had the same composition as those reported thus far^9^.

**Figure 5:**
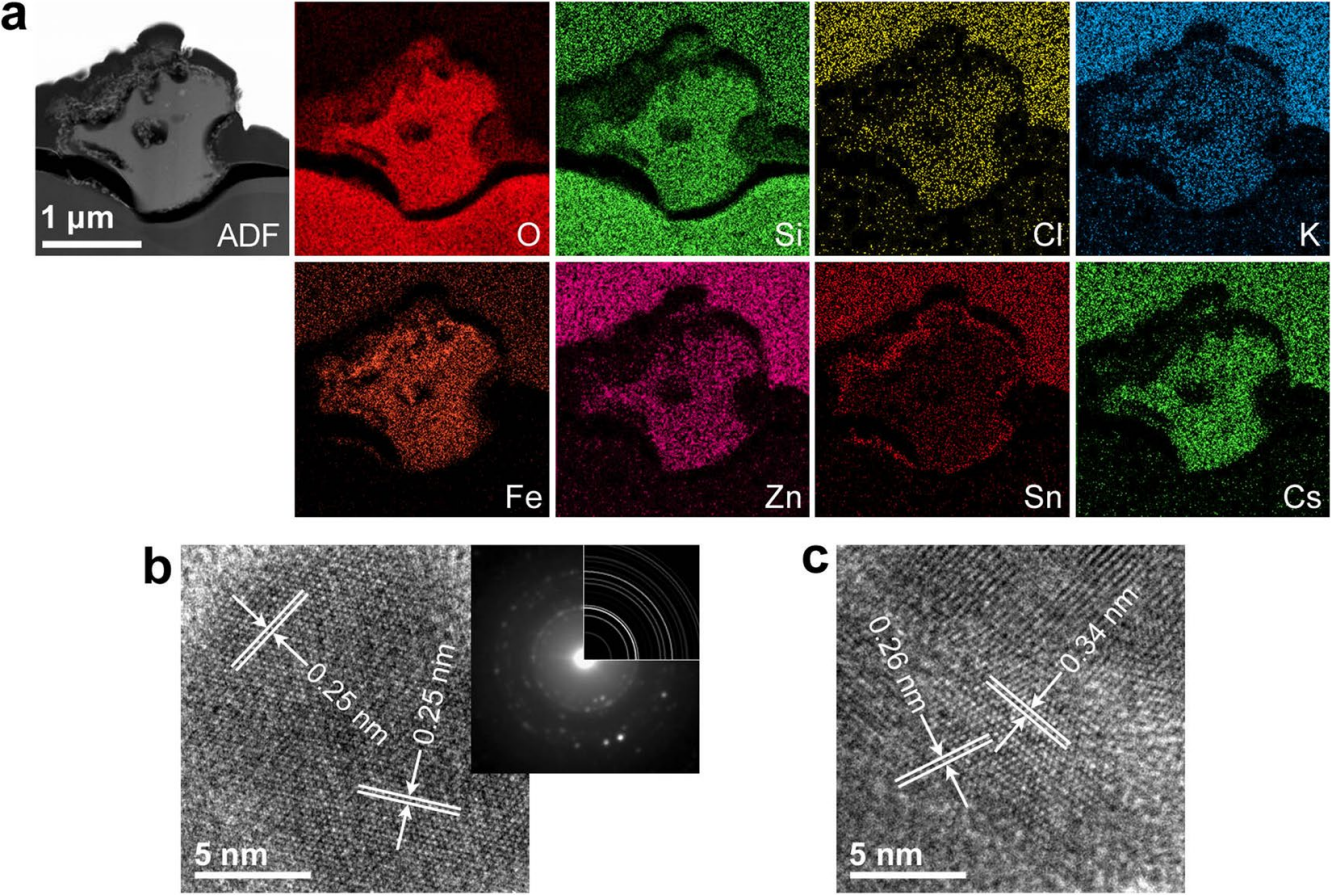
TEM/STEM analysis of PS120-2 after the dissolution experiment with pure water. (**a**) STEM-ADF image and corresponding element maps. (**b**) High-resolution TEM image of Fe-rich precipitates, showing lattice fringes of 0.25 nm. The upper-right inset is an electron diffraction pattern acquired from the precipitates and a Debye-Scherrer pattern of maghemite. (**c**) High-resolution TEM image of Sn-rich precipitates, showing lattice fringes of 0.26 and 0.34 nm, which correspond to cassiterite.

In the case of the CsMPs dissolved in pure water, a large hole formed in PS90-3, which was consistent with the SEM image (Fig. S6). If the dissolution had continued, this particle might have split into several fragments. For the CsMPs that could not be recovered, the particles were likely broken into several fragments, as implied by the image shown in Fig. S6. The shape of PS120-2 after the dissolution experiment included several concave areas (Fig. 5a), which were not observed in the particle before dissolution by SEM. Moreover, several holes formed inside the particle in the STEM-annular dark-field (ADF) image, which suggested that the dissolution proceeded inhomogeneously and intricately. The elemental maps of Fe and Sn for PS120-2 indicated that these elements were enriched around the surface of the particle, and nanoparticulates were observed in the corresponding regions in the STEM-ADF image. In addition to PS120-2, Sn-enriched particulates were observed around the surface of PS90-3 (Fig. S6). The Fe-rich particulates in PS120-2 showed lattice fringes of 0.25 nm in the high-resolution TEM image, and their electron diffraction patterns indicated a spinel structure, which suggested that maghemite (γ-Fe_2_O_3_) precipitated around the CsMPs (Fig. 5b). The Sn-rich particulates showed lattice fringes of 0.26 and 0.34 nm in the high-resolution TEM image (Fig. 5c), corresponding to cassiterite (SnO_2_). These nanoparticulates likely precipitated during dissolution because of the low solubility of Sn^4+^ and Fe^3+^.

**Figure 6:**
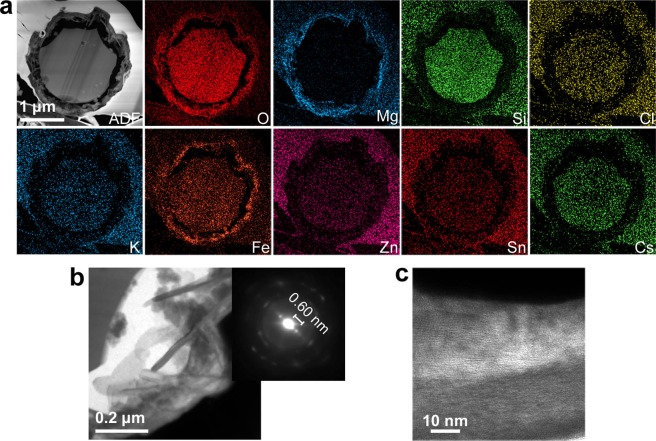
TEM/STEM analysis of SS90-2 after the dissolution experiment with seawater. (**a**) STEM-ADF image and corresponding element maps. (**b**) Bright-field TEM image of the plate-like materials rich in Mg and Fe that formed between the surface and the glass matrix. The upper-right inset is an electron diffraction pattern acquired from the plate-like materials. (**c**) High-resolution TEM image of the plate-like materials.

In the case of the CsMPs (SS90-2) dissolved in seawater, a crust of secondary minerals rich in Mg and Fe formed on the surface according to the STEM-EDS analysis (Fig. 6a). Although the size of the CsMPs did not appear to change in the SEM images, the glass matrix of the CsMPs decreased inside the crust due to dissolution, resulting in a decrease in the ^137^Cs radioactivity. The TEM images and electron diffraction patterns showed that the secondary minerals in the crust consisted of plate-like crystals with a layer spacing of 0.60 nm (Fig. 6b,c). The crust might be an altered layer that formed due to hydrolysis of the glass matrix. Although another CsMP dissolved in seawater also had a surface crust rich in Mg and Fe, the outermost surface was enriched in Sn (Fig. S7). As mentioned above, the Sn-rich layer was created by the dissolution of CsMPs in pure water. In addition, the Sn-rich layer has been reported in previous research on CsMPs collected from the environment^8–10^. Therefore, the Sn-rich layer in SS90-1 might be formed in the environment, and when the CsMPs dissolved in seawater, secondary minerals rich in Mg and Fe precipitated inside the outermost Sn-rich layer. The glass matrix was dissolved because the layers of secondary minerals were not so complete that seawater could pass through them. In the narrow space between the outermost layer and glass matrix, Mg and Fe were saturated more easily and secondary minerals precipitated there.

## Discussion

When CsMPs were first found on aerosol filters, Adachi *et al*.^6^ reported that they were a kind of alloy and insoluble in water, unlike other aerosols such as sulfates, which were considered to be the major carriers of radiocaesium^3^. However, Yamaguchi *et al*.^8^ reported that CsMPs are mainly composed of silicate glass, which implies that CsMPs are subject to weathering in the field and that their radioactivity may be reduced by processes other than radiocaesium decay, such as dissolution and leaching. Generally, the dissolution rate of silicate glass significantly varies depending on its composition. As reported by Yamaguchi *et al*.^8^ and Kogure *et al*.^9^, CsMPs contain considerable amounts of Cs, Zn, and Fe, while Na, Ca and Al are absent. Such a composition is not common for silicate glass, and its dissolution rate has not been investigated thus far. According to Perera *et al*.^19^, the dissolution rate of silicate glass in water (pH = 7) at 25 °C varies from 10^−3^ µm/y for obsidian glass (natural volcanic glass) to approximately 5 µm/y for soda-lime glass with a composition of 72SiO_2_-22Na_2_O-6CaO. Our results suggested that the dissolution rate of CsMPs in pure water and seawater is on the order of 10^−2^ and 10^−1^ µm/y, respectively, at 25 °C, which is relatively low but not as low as that of obsidian glass. For the comparison with other previous works, the dissolution rate at 100 °C was calculated and converted into 0.06 and 6 g/m^2^/d for pure water and seawater, respectively, using the density of CsMPs (2.6 g/cm^3^)^12^. These values are comparable with those of some nuclear waste glasses although the experiment conditions are not exactly the same^20–22^. Besides glass composition, the glass-surface-to-solution-volume (*S*/*V*) ratio and glass surface conditions like the formation of a protective layer are known to influence the dissolution rate^20,22–24^. In the present study, the CsMPs of several micron size were immersed in 20 mL aqueous solutions. Accordingly, the *S*/*V* ratio was less than 10^−5^ m^−1^ and the solution condition was far from equilibrium through the experiments. The possibility of the protective layer formation is also excluded because the dissolution rate did not decrease with the incubation time.

The dissolution experiments in this study revealed that CsMPs are dissolved approximately 10 times faster in seawater than in pure water at 13 °C. CsMPs will take decades to completely dissolve in water with a pH of approximately 5, whereas CsMPs will dissolve in only several years in seawater with a mean temperature of approximately 13 °C. Considering that CsMPs are intricately dissolved without maintaining a spherical shape, the dissolution may proceed more rapidly because the surface area of CsMPs increases. Thus, almost all CsMPs that fell into the sea immediately after the FDNPP accident may have already dissolved at present as more than seven years have passed since the accident. Additionally, if CsMPs are transported to the sea through rivers, their dissolution is considerably accelerated. In general, the dissolution rate of silicate glass is the slowest under slightly acidic conditions and becomes faster with decreasing and increasing pH^25,26^. According to previous research^25^, soda-lime glass dissolves approximately five times faster at pH = 8.3 than 5.2 in a NaCl solution at 75 °C. Accordingly, the difference in the dissolution rate of CsMPs in the two solutions in this study may be largely owing to the difference in pH (5.2 and 8.3 for pure water and seawater, respectively). In addition to pH, dissolved ions in seawater might influence the glass dissolution. For instance, the presence of Mg is known to promote the glass dissolution because Mg removes silica from glass and precipitates as a secondary mineral^21,27,28^.

Yamaguchi *et al*.^10^ reported the structure of a CsMP (named “B-1” in their paper) collected in July 2015, approximately four years after the FDNPP accident, from the atmosphere using a high-volume air sampler. This CsMP had a rectangular shape, and Sn- and Fe-rich materials had formed on its surface. Such structural features resembled those observed in PS120-2 (Fig. 5), which suggested that B-1 in Yamaguchi *et al*.^10^ experienced partial dissolution, similar to the CsMPs in this study, in the field during the four year period. On the other hand, Yamaguchi *et al*.^8^ also found an alkali-depleted crust in the vicinity of the surface of a CsMP collected from Japanese cedar leaves. Such a crust was not formed in the dissolution experiments in this study. An alkali-depleted crust in alkali-bearing silicate glass is known to form if the pH of the reaction solution is low^29^; thus, it was not observed under the dissolution conditions in this study.

The present study is the first to investigate the lifetime of CsMPs. The efficient collection of many CsMPs enabled dissolution experiments to be performed to estimate the dissolution rate. Additionally, the dissolution behaviour of CsMPs was revealed by electron microscope observations of CsMPs before and after dissolution. As the next step, the dissolution behaviour of CsMPs in interstitial water in soil, lacustrine deposits, bodily fluid and so on should be investigated for a comprehensive discussion of the fates of CsMPs and the influence of various environments on their radioactivity at present and in the future.

## Methods

### Collection of CsMPs

CsMPs were collected from NWC laid on a vegetable field in Fukushima Prefecture. The NWC was left outside for approximately six months after the FDNPP accident. The NWC was then cut into fragments of 15 × 15 mm and immersed in ion-exchanged water for 12 h to remove the easily soluble form of radiocaesium. The NWC fragments were exposed to an IP (BAS-2500, Fujifilm) for 10 min to confirm the presence of CsMPs as bright spots in the readout images of the IP.

The isolation of individual CsMPs was carried out using a previously described method^15^. The CsMPs attached to the NWC were transferred to 10 mL of ion-exchanged water by ultrasonication. After removing the NWC from the water, the water was divided into 0.5 mL aliquots, and aliquots of water containing CsMPs were identified by measuring the radioactivity with an automatic gamma counter (Wizard2480, PerkinElmer). Ten millilitres of ion-exchanged water were added to each selected aliquot of water, and these aliquots were again divided into 20 aliquots of 0.5 mL. The aliquots containing CsMPs were identified again. This process was repeated several times to isolate individual CsMPs from other unrelated particles. The water suspending the CsMP was dropped onto a plastic plate previously coated with carbon and dried at ambient temperature. The CsMPs were observed using a Hitachi S-4500 SEM equipped with a Kevex Sigma EDS spectrometer, and their radioactivity was determined using a germanium semiconductor gamma-ray spectrometer (GCW2523S, Canberra). The values of ^137^Cs radioactivity were corrected to those on 14 March 2011. The detection efficiency was calibrated by a point-source standard prepared from filter paper with 100 Bq of ^137^Cs onto which a ^137^Cs solution (CZ005 Japan Radioisotope Association, Tokyo, Japan) was dropped and dried.

### Single CsMP dissolution experiment

Each identified CsMP was transferred to 20 mL of pure water or seawater and incubated at temperatures of 90 °C and 120 °C. Ion-exchanged water and Daigo’s Artificial Seawater SP (Nihon Pharmaceutical) were used as the pure water and artificial seawater, respectively. During incubation, the water phase (19.5 mL) was replaced periodically (120 h and 12 h at 90 °C and 120 °C, respectively) after allowing the reaction bottles to stand for 12 h so the CsMPs could sink to the bottom of the bottles. The water phase was periodically removed from the bottles, and the radioactivity of ^137^Cs dissolved in the water phase was measured using a germanium detector. The detection efficiency for the solution analyses was calibrated with a ^137^Cs standard solution supplied by the Japan Radioisotope Association (CZ005 Japan Radioisotope Association, Tokyo, Japan). The incubation period was continued until the radioactivity of ^137^Cs in the CsMPs decreased to approximately half the original radioactivity level. After the final incubation period, the water phase and CsMPs were separated, and the CsMPs were transferred onto Kapton tape for the following electron microscopy analyses.

SEM observations and analyses of the elemental composition of the CsMPs after the dissolution experiments were conducted. After the SEM analyses, the CsMPs were thinned to an electron-transparent thickness using an FIB system with a micro-sampling unit (Hitachi FB-2100). STEM-EDS analysis of these thin specimens was conducted using a JEOL JEM-2800 STEM operated at 200 kV with an X-Max^N^ 100TLE silicon drift detector (Oxford Instruments). Nanoparticulates around the surface of the CsMPs were also identified using a JEOL JEM-2010 TEM operated at 200 kV.

### Multiple CsMP dissolution experiment

The NWC fragments with more than five bright spots identified by IP autoradiography were selected. They were tightly folded to 2 × 2 mm in size, and the radioactivity of ^137^Cs was determined by a germanium detector. After the measurement, the fragments of NWC were wrapped with a hydrophilic Teflon filter (pore size 0.1 μm, H010A025A, Advantec) to prevent CsMPs from detaching from the NWC. The wrapped NWC fragments with multiple CsMPs were immersed in 20 mL of pure water or seawater and then incubated under three conditions, namely, 30 °C, 60 °C, and 90 °C. After incubation, the water phase was transferred to a plastic bottle, and the radioactivity of ^137^Cs dissolved in the water phase was determined.

## Supplementary information

Supplementary information

## Data Availability

The data that support the findings of this study are available from the corresponding author upon reasonable request.
