# Supplementary material for: Dissolution behaviour of radiocaesium-bearing microparticles released from the Fukushima nuclear plant
Source: Sci Rep. 2019 Mar 5;9:3520. doi: 10.1038/s41598-019-40423-x (PMC6401112; doi:10.1038/s41598-019-40423-x)
Supplement: Supplementary file 1 — Supplementary information [file 41598_2019_40423_MOESM1_ESM.docx]

Supplementary Information for

Dissolution behaviour of radiocaesium-bearing microparticles
released from the Fukushima nuclear plant

Taiga Okumura^1,^*, Noriko Yamaguchi^2^ Terumi Dohi^3^, Kazuki Iijima^3^ and Toshihiro Kogure^1^

^1^Department of Earth and Planetary Science, Graduate School of Science, The University of Tokyo, 7-3-1 Hongo, Bunkyo-ku, Tokyo 113-0033, Japan

^2^Institute for Agro-Environmental Sciences, NARO, 3-1-3 Kannondai, Tsukuba, Ibaraki 305-0864, Japan

^3^Fukushima Environmental Safety Center, Sector of Fukushima Research and Development, Japan Atomic Energy Agency, 10-2 Fukasaku, Miharu-machi, Tamura-gun, Fukushima, 963-7700, Japan

*Corresponding author

E-mail: okumura@eps.s.u-tokyo.ac.jp Tel: +81-3-5841-4545

# Results

Since some radiocaesium-bearing microparticles (CsMPs) have been reported to have a radial distribution of Cs with a higher concentration near the surface, the dissolution rate of CsMPs was recalculated assuming a radial distribution of Cs similar to that of the CsMP designated P6-6 in the previous research.^1^ Figure S2 shows the average X-ray counts of Cs in P6-6 (as open circle symbols), measured using energy-dispersive X-ray spectrometry (EDS), as a function of the radius. In this CsMP, the concentration of Cs drastically decreased approximately 0.3 µm from the surface and then was constant in the inner part of the particle. This EDS spectrum of Cs was fitted with a Gaussian function as follows:

|  | $I\left( r \right)=77\exp\left( -\frac{4\ln\left( 2 \right)\left( r-r_{0} \right)^{2}}{{0.36}^{2}} \right)+14$ | (S1) |
| --- | --- | --- |

where *I*(*r*) is the X-ray counts of Cs at a radius *r* (µm). In the same manner as P6-6, the Cs in the CsMPs used in this study was assumed to be concentrated in the approximately 0.3 µm thick surface layer, and the radioactivity of ^137^Cs corresponding to the radius can be expressed as follows:

|  | $C\left( r \right)=AI\left( r \right)$ | (S2) |
| --- | --- | --- |

where *C*(*r*) is the radioactivity of ^137^Cs at a radius *r*, and *A* is a coefficient unique to each CsMP. In other words, the distribution of ^137^Cs radioactivity in CsMPs was assumed to follow the above Gaussian function with a full-width at half-maximum of 0.36 µm. The coefficient *A* was determined so that the integral of 4π*r*^2^*C*(*r*) corresponded to the original radioactivity, *R*_0_. Then, the radius of the CsMPs at which the integral of 4π*r*^2^*C*(*r*) was equal to the radioactivity, *R*, during the dissolution experiments was determined and is shown in Table S1–S4 as *r*_i_. Using Equation (3) and *r*_i_, the decrease in the CsMP radius (*r*_0_ − *r*) is shown as a function of the incubation time (*t*) in Fig. S3. The decrease in the CsMP radius was approximately proportional to the incubation time with the same trend observed in Fig. 1, but the radii values were approximately half the values obtained with the assumption of a homogeneous Cs distribution. Note that the vertical axis in Fig. S3 is different from that in Fig. 1. Figure S4 shows the Arrhenius plot (logarithm of *k* versus the reciprocal temperature, 1/*T*) constructed from the *k* obtained from the slopes in Fig. S3. The R^2^ value of the regression line was 0.92 and 0.98 for pure water and seawater, respectively. The activation energy was calculated to be 65 and 92 kJ/mol for pure water and seawater, respectively. Additionally, the rate of decrease in the radius of the CsMPs (*k*) was estimated to be 0.007 and 0.058 µm/y for pure water and seawater, respectively, at 13 °C. Accordingly, the dissolution rate of the CsMPs was approximately half the rate obtained with the assumption of a homogeneous Cs distribution.

# Supplementary Tables

**Table S1.** Change in the radioactivity (^137^Cs) and radius of radiocaesium-bearing microparticles (CsMPs) in the single CsMP experiment with pure water

| Sample | Temp. (°C) | Time (h) | ^137^Cs (Bq) | *r*_h_ (μm) | *r*_i_ (μm) |
| --- | --- | --- | --- | --- | --- |
| PS120-1 | 120 | 0 | 1.45 | 1.00 | 1.00 |
|  |  | 12 | 1.37 | 0.98 | 0.99 |
|  |  | 24 | 1.28 | 0.96 | 0.98 |
|  |  | 36 | 1.14 | 0.92 | 0.96 |
|  |  | 48 | 0.99 | 0.88 | 0.94 |
|  |  | 60 | 0.83 | 0.83 | 0.91 |
|  |  | 72 | 0.70 | 0.79 | 0.89 |
| PS120-2 | 120 | 0 | 3.93 | 1.27 | 1.27 |
|  |  | 12 | 3.79 | 1.25 | 1.26 |
|  |  | 36 | 3.54 | 1.23 | 1.25 |
|  |  | 60 | 3.41 | 1.21 | 1.24 |
|  |  | 132 | 3.03 | 1.17 | 1.22 |
|  |  | 299 | 2.26 | 1.06 | 1.17 |
|  |  | 467 | 1.37 | 0.89 | 1.10 |
| PS120-3 | 120 | 0 | 1.77 | 1.09^a^ | 1.09^a^ |
|  |  | 72 | 1.13 | 0.94 | 1.02 |
|  |  | 120 | 0.85 | 0.85 | 0.98 |
| PS90-1 | 90 | 0 | 0.91 | 0.87^a^ | 0.87^a^ |
|  |  | 120 | 0.91 | 0.87 | 0.87 |
|  |  | 360 | 0.63 | 0.77 | 0.82 |
|  |  | 600 | 0.47 | 0.70 | 0.78 |
|  |  | 696 | 0.43 | 0.68 | 0.77 |
| PS90-2 | 90 | 0 | 0.86 | 0.86^a^ | 0.86^a^ |
|  |  | 120 | 0.76 | 0.82 | 0.84 |
|  |  | 360 | 0.49 | 0.71 | 0.77 |
|  |  | 600 | 0.26 | 0.57 | 0.70 |
| ^a^These values were estimated from PS120-1, PS120-2, SS90-1, SS90-2, and SS90-3. | | | | | |

**Table S2.** Change in the radioactivity (^137^Cs) and radius of CsMPs in the multiple CsMP experiment with pure water

| Sample | Temp. (°C) | Time (d) | ^137^Cs (Bq) | *r*_h_ (μm) | *r*_i_ (μm) |
| --- | --- | --- | --- | --- | --- |
| PM90-1 | 90 | 0 | 6.30 | 1.16 | 1.16 |
|  |  | 2 | 5.52 | 1.11 | 1.14 |
| PM90-2 | 90 | 0 | 5.05 | 1.16 | 1.16 |
|  |  | 2 | 4.18 | 1.09 | 1.13 |
| PM90-3 | 90 | 0 | 9.43 | 1.16 | 1.16 |
|  |  | 2 | 7.80 | 1.09 | 1.13 |
| PM60-1 | 60 | 0 | 10.74 | 1.16 | 1.16 |
|  |  | 51 | 7.42 | 1.03 | 1.10 |
| PM60-2 | 60 | 0 | 11.07 | 1.16 | 1.16 |
|  |  | 51 | 8.92 | 1.08 | 1.12 |
| PM60-3 | 60 | 0 | 12.50 | 1.16 | 1.16 |
|  |  | 51 | 9.85 | 1.07 | 1.12 |
| PM30-1 | 30 | 0 | 13.57 | 1.16 | 1.16 |
|  |  | 250 | 12.30 | 1.13 | 1.14 |
| PM30-2 | 30 | 0 | 9.57 | 1.16 | 1.16 |
|  |  | 250 | 8.69 | 1.13 | 1.14 |
| PM30-3 | 30 | 0 | 6.42 | 1.16 | 1.16 |
|  |  | 250 | 5.89 | 1.13 | 1.15 |

**Table S3.** Change in the radioactivity (^137^Cs) and radius of CsMPs in the single CsMP experiment with sea water

| Sample | Temp. (°C) | Time (h) | ^137^Cs (Bq) | *r*_h_ (μm) | *r*_i_ (μm) |
| --- | --- | --- | --- | --- | --- |
| SS90-1 | 90 | 0 | 1.12 | 1.07 | 1.07 |
|  |  | 8 | 0.15 | 0.55 | 0.78 |
| SS90-2 | 90 | 0 | 3.31 | 1.32 | 1.32 |
|  |  | 12 | 0.70 | 0.78 | 1.06 |
| SS90-3 | 90 | 0 | 1.73 | 1.16 | 1.16 |
|  |  | 12 | 0.16 | 0.53 | 0.77 |

**Table S4.** Change in the radioactivity (^137^Cs) and radius of CsMPs in the multiple CsMP experiment with seawater

| Sample | Temp. (°C) | Time (h) | ^137^Cs (Bq) | *r*_h_ (μm) | *r*_i_ (μm) |
| --- | --- | --- | --- | --- | --- |
| SM90-1 | 90 | 0 | 2.26 | 1.16 | 1.16 |
|  |  | 6 | 1.48 | 1.01 | 1.09 |
|  |  | 12 | 0.84 | 0.84 | 1.01 |
|  |  | 18 | 0.46 | 0.69 | 0.92 |
| SM90-2 | 90 | 0 | 2.24 | 1.16 | 1.16 |
|  |  | 6 | 0.70 | 0.79 | 0.98 |
|  |  | 12 | 0.23 | 0.55 | 0.79 |
|  |  | 18 | 0.11 | 0.43 | 0.63 |
|  |  | 24 | 0.08 | 0.38 | 0.56 |
| SM90-3 | 90 | 0 | 4.29 | 1.16 | 1.16 |
|  |  | 6 | 1.37 | 0.79 | 0.99 |
|  |  | 12 | 0.76 | 0.65 | 0.90 |
|  |  | 18 | 0.50 | 0.57 | 0.82 |
|  |  | 24 | 0.37 | 0.51 | 0.76 |
|  |  | 30 | 0.28 | 0.47 | 0.69 |
| SM60-1 | 60 | 0 | 4.18 | 1.16 | 1.16 |
|  |  | 12 | 3.69 | 1.11 | 1.14 |
|  |  | 24 | 3.08 | 1.05 | 1.11 |
|  |  | 36 | 2.63 | 1.00 | 1.08 |
|  |  | 48 | 2.01 | 0.91 | 1.04 |
|  |  | 60 | 1.63 | 0.85 | 1.01 |
|  |  | 72 | 1.37 | 0.80 | 0.99 |
|  |  | 84 | 1.12 | 0.75 | 0.96 |
|  |  | 96 | 0.91 | 0.70 | 0.93 |
| SM60-2 | 60 | 0 | 1.75 | 1.16 | 1.16 |
|  |  | 12 | 1.68 | 1.15 | 1.15 |
|  |  | 24 | 1.59 | 1.13 | 1.14 |
|  |  | 36 | 1.41 | 1.08 | 1.12 |
|  |  | 48 | 1.18 | 1.02 | 1.09 |
|  |  | 60 | 1.01 | 0.97 | 1.07 |
|  |  | 72 | 0.87 | 0.92 | 1.05 |
|  |  | 84 | 0.72 | 0.87 | 1.02 |
|  |  | 96 | 0.60 | 0.82 | 1.00 |
| SM60-3 | 60 | 0 | 2.58 | 1.16 | 1.16 |
|  |  | 12 | 2.28 | 1.12 | 1.14 |
|  |  | 24 | 2.06 | 1.08 | 1.12 |
|  |  | 36 | 1.80 | 1.03 | 1.10 |
|  |  | 48 | 1.60 | 0.99 | 1.08 |
|  |  | 60 | 1.42 | 0.95 | 1.06 |
|  |  | 72 | 1.27 | 0.92 | 1.05 |
|  |  | 84 | 1.03 | 0.86 | 1.02 |
|  |  | 96 | 0.88 | 0.81 | 0.99 |
| SM30-1 | 30 | 0 | 1.92 | 1.16 | 1.16 |
|  |  | 336 | 1.74 | 1.12 | 1.14 |
|  |  | 1200 | 1.45 | 1.06 | 1.11 |
|  |  | 2400 | 1.05 | 0.95 | 1.06 |
| SM30-2 | 30 | 0 | 1.86 | 1.16 | 1.16 |
|  |  | 336 | 1.72 | 1.13 | 1.15 |
|  |  | 1200 | 1.27 | 1.02 | 1.10 |
|  |  | 2400 | 0.88 | 0.91 | 1.04 |
| SM30-3 | 30 | 0 | 1.68 | 1.16 | 1.16 |
|  |  | 336 | 1.51 | 1.12 | 1.14 |
|  |  | 1200 | 1.19 | 1.04 | 1.10 |
|  |  | 2400 | 0.60 | 0.83 | 1.00 |

**Table S5.** Chemical composition (wt.%) of the CsMPs designated PS120-2 and SS90-1

| Sample | PS120-2 | SS90-1 |
| --- | --- | --- |
| SiO_2_ | 69.7 | 78.7 |
| Cl | 1.1 | 0.5 |
| K_2_O | 1.0 | 2.2 |
| MnO | 0.6 | 0.3 |
| Fe_2_O_3_ | 12.7 | 6.0 |
| ZnO | 3.1 | 8.1 |
| Rb_2_O | 0.3 | 1.8 |
| SnO_2_ | 1.8 | 1.3 |
| Cs_2_O | 9.7 | 1.1 |
| Total | 100.0 | 100.0 |

# Supplementary Figures


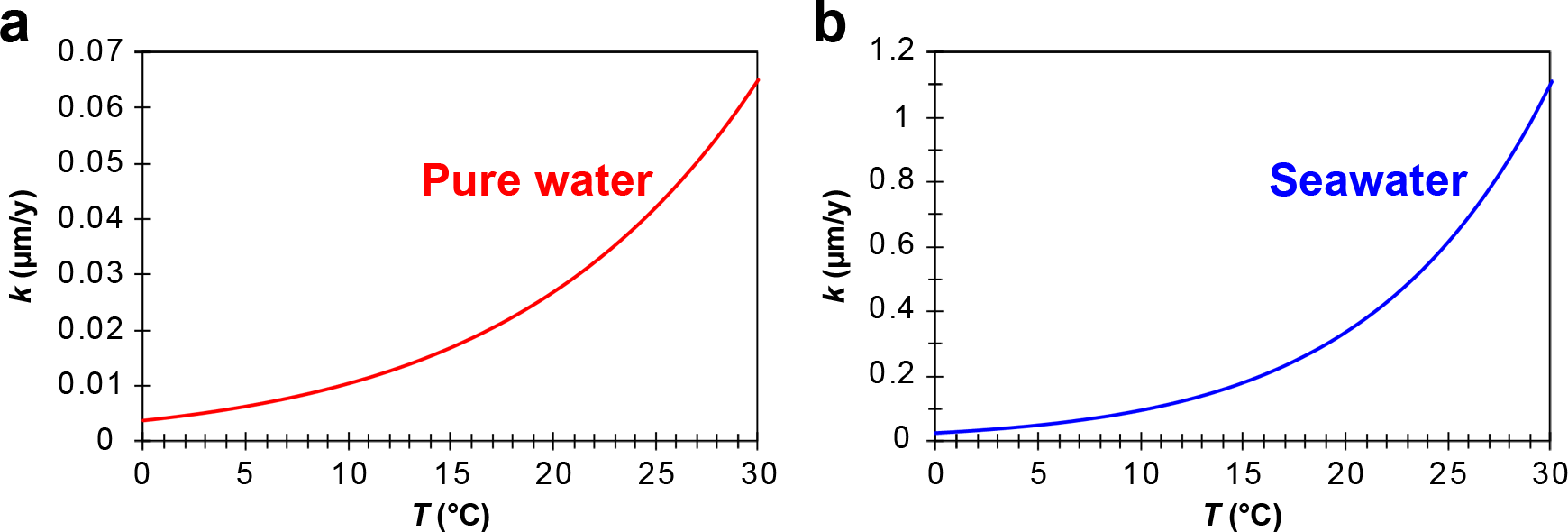


**Figure S1.** Rate of decrease in the radius of CsMPs (*k*) versus temperature (*T*) assuming homogeneous distribution of Cs in the CsMPs. (a) Pure water. (b) Seawater.


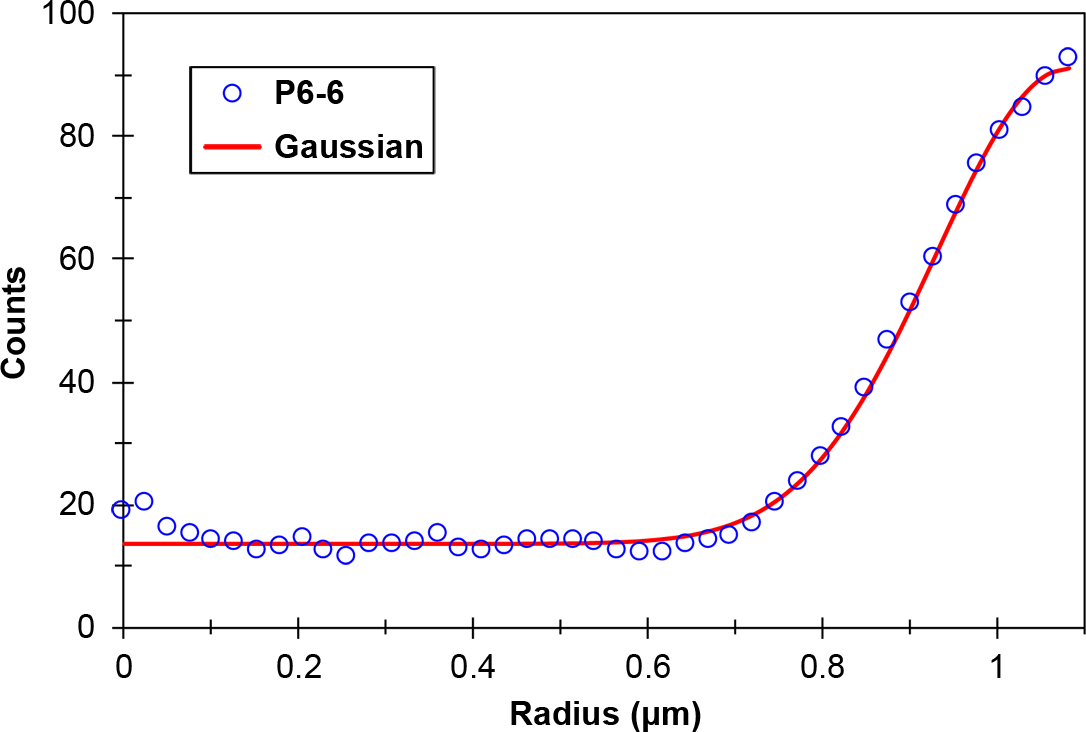


**Figure S2.** Average X-ray intensity of Cs measured by energy-dispersive X-ray spectroscopy (EDS) versus the radius of the CsMP designated P6-6^1^ and its Gaussian fitting.


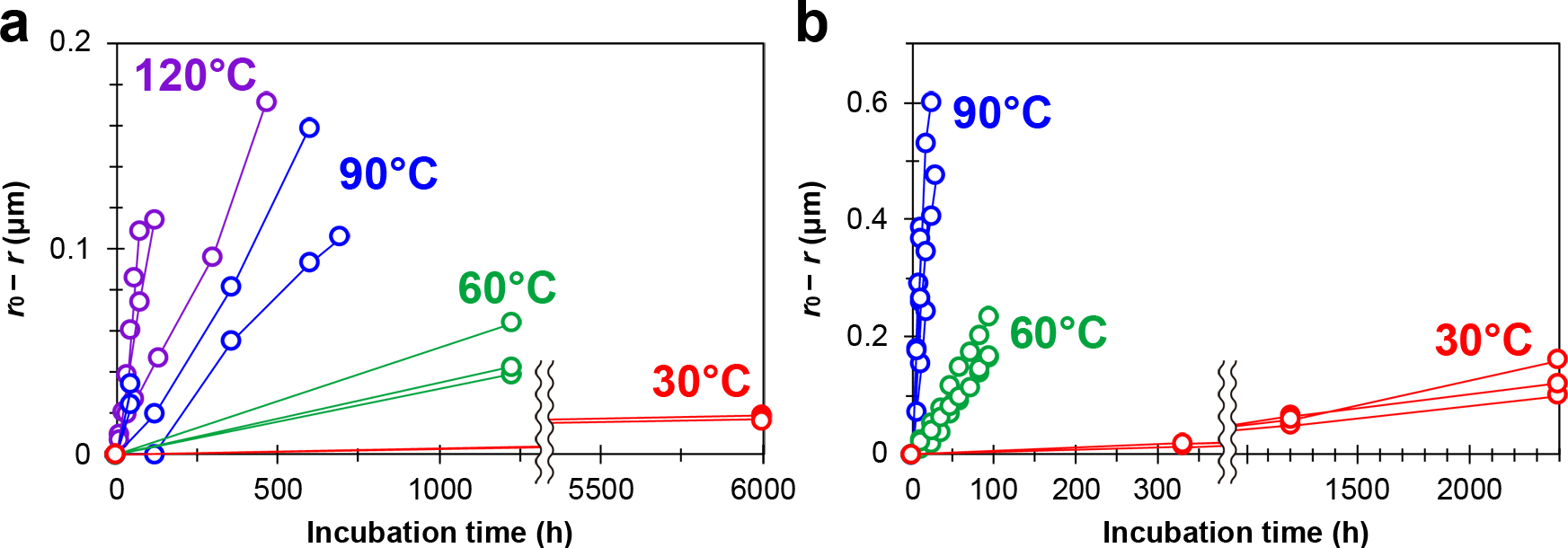


**Figure S3.** Decrease in the CsMP radius (*r*_0_ − *r*) versus incubation time assuming a Gaussian distribution of Cs in the CsMPs. (a) Pure water. (b) Seawater.


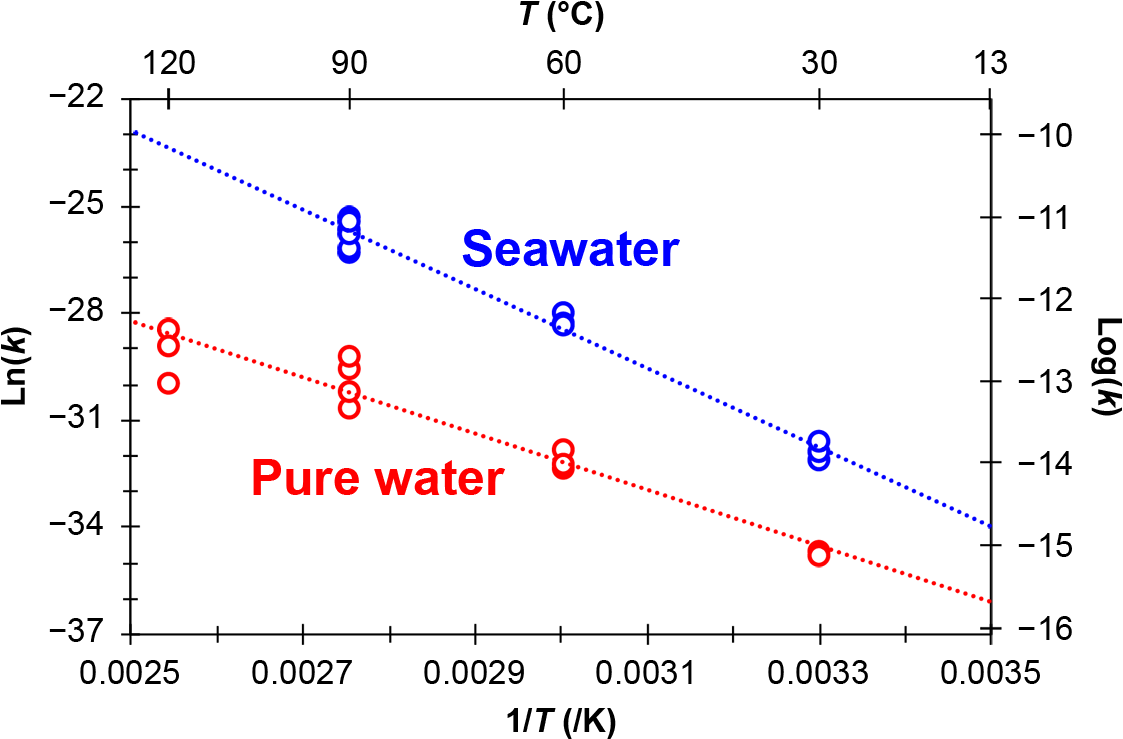


**Figure S4.** Arrhenius plot of logarithm *k* (rate of decrease in the radius of the CsMPs; m/s) versus the reciprocal temperature, 1/*T*, assuming a Gaussian distribution of Cs in the CsMPs.


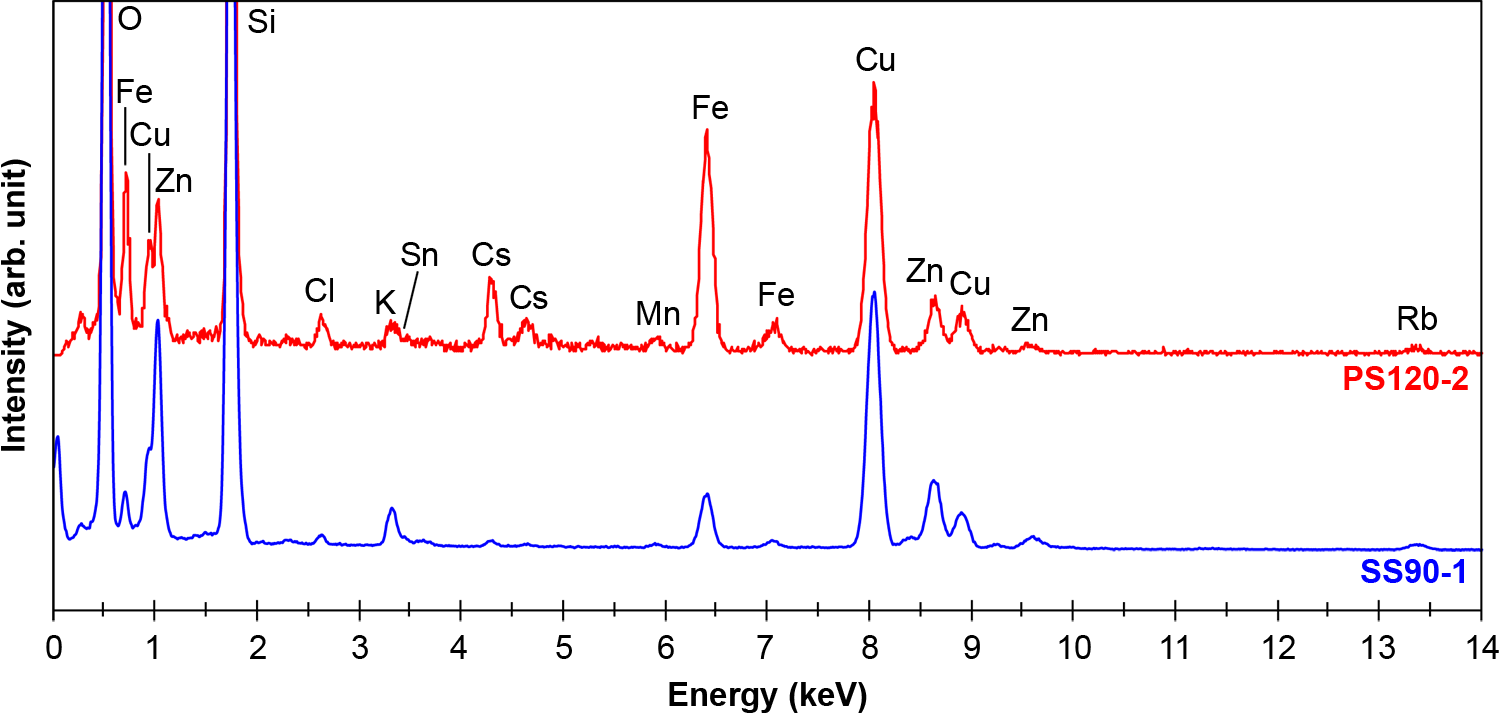


**Figure S5.** EDS spectra acquired from the centre of the CsMPs of PS120-2 and SS90-1. Cu peaks are from a grid supporting the specimen.


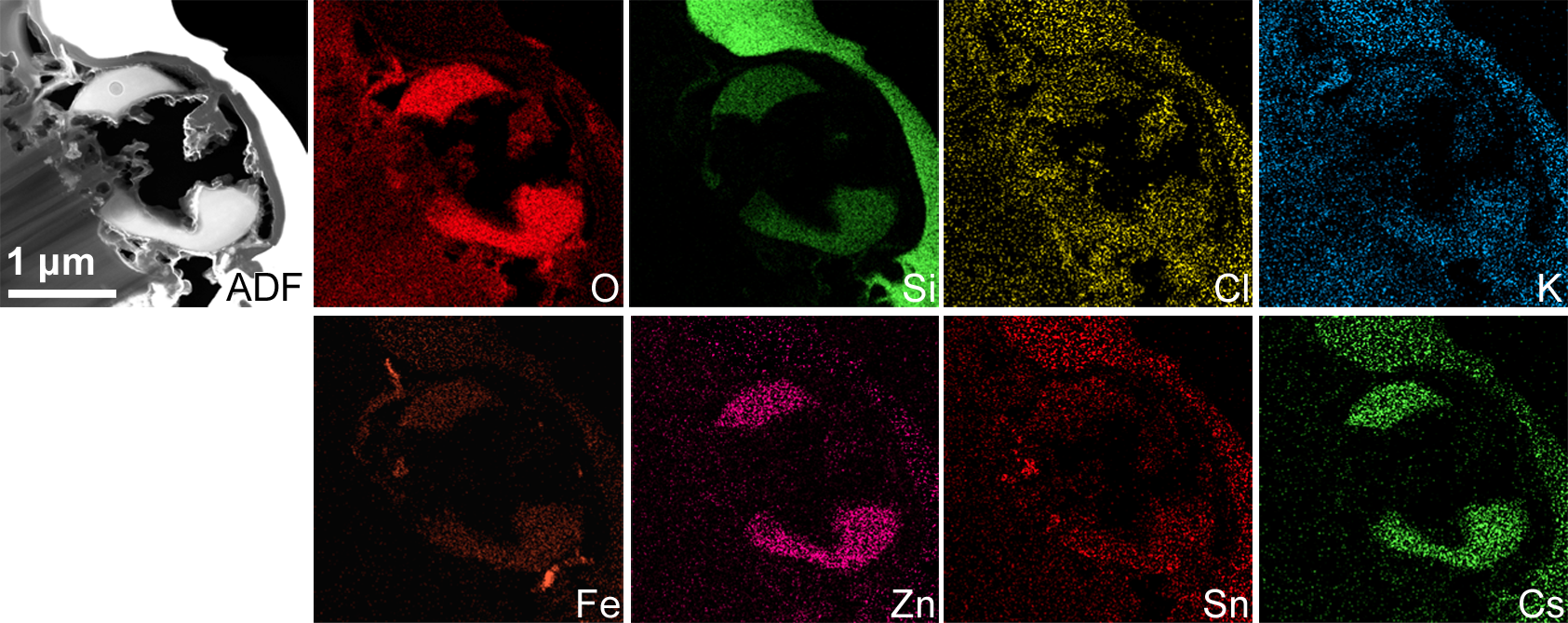


**Figure S6.** Scanning transmission electron microscopy-annular dark-field (STEM-ADF) image and corresponding element maps of PS90-3 after the dissolution experiment in pure water.


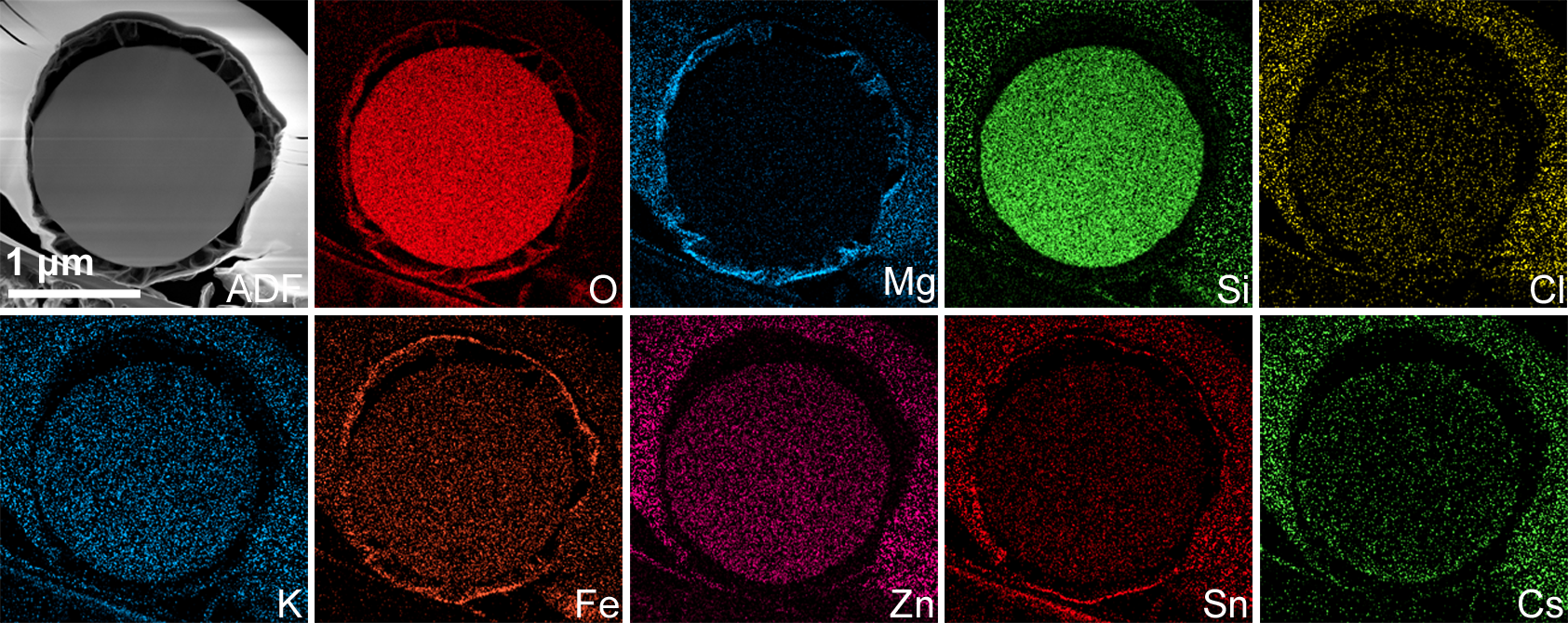


**Figure S7.** STEM-ADF image and corresponding element maps of SS90-1 after the dissolution experiment in seawater.

# References

1. Kogure, T. *et al.* Constituent elements and their distribution in the radioactive Cs-bearing silicate glass microparticles released from Fukushima nuclear plant. *Microscopy* **65,** 451–459 (2016).
